# Complete genome sequence of *Dorea formicigenerans* JCM 31256

**DOI:** 10.1128/mra.01254-24

**Published:** 2024-12-27

**Authors:** Dieter M. Tourlousse, Kazutoshi Murotomi, Mayu Hamajima, Yuji Sekiguchi

**Affiliations:** 1 Biomedical Research Institute, National Institute of Advanced Industrial Science and Technology (AIST), Tsukuba, Japan; Wellesley College Department of Biological Sciences, Wellesley, Massachusetts, USA

**Keywords:** *Dorea formicigenerans*, complete genome, Oxford Nanopore Technologies

## Abstract

We describe a complete genome sequence of *Dorea formicigenerans* JCM 31256. The genome consists of a single circular chromosome with a length of 3,090,452 base pairs and a GC content of 40.8%, and was predicted to contain 3,061 total genes, encoding for 2,907 proteins.

## ANNOUNCEMENT

*Dorea formicigenerans*, previously known as *Eubacterium formicigenerans* (1, 2), is a Gram-positive anaerobic bacterium and common commensal in the human gut. *D. formicigenerans* has a fermentative metabolism and was named after its ability to produce copious amounts of formic acid (1, 2). Although its role in human health and disease remains poorly understood, an elevated abundance of *D. formicigenerans* in overweight/obese human subjects has been reported (3). In another study, Cox et al. (4) showed that the abundance of *D. formicigenerans* was decreased in the feces of patients with progressive and relapsing-remitting multiple sclerosis. Here, we employed nanopore sequencing to obtain a complete genome sequence of *D. formicigenerans* JCM 31256, a strain previously isolated from human feces.

Strain JCM 31256 was obtained as liquid-dried (L-dried) cells from the Japan Collection of Microorganisms and cultured in yeast casitone fatty acid (YCFA) broth under an atmosphere of N_2_/CO_2_ [80/20 (vol/vol)] at 37°C for ~20 hrs. High-molecular-weight DNA was extracted from the overnight-grown culture using the Nanobind CBB kit RT (PacBio). DNA was prepared for sequencing using the Native Barcoding kit (SQK-NBD114.24; barcode NB02) from Oxford Nanopore Technologies (ONT), without fragmentation and following the manufacturer’s protocol. Sequencing was performed on an R10.4.1 flow cell using a GridION device (MinKNOW v24.02.16), with default sequencing parameters (speed, 400 bases per second; sampling frequency, 5 kHz). Bioinformatics procedures below used default settings unless specified. Basecalling was performed with Dorado v0.8.2 (https://github.com/nanoporetech/dorado) using model dna_r10.4.1_e8.2_400bps_sup@v5.0.0. Low-quality reads (mean quality of <16) and short reads (length of <1,000 or <5,000 bases) were discarded using fastq-filter v0.3.0 (https://github.com/LUMC/fastq-filter). The reads with a length of >5,000 bases (141,022 reads and 2,009,208,248 bases with an N50 of 17,537; ~650× coverage) were split into nine minimally overlapping subsets using Trycycler v0.5.5’s (5) subsample command. The subsampled reads (~18,000 reads and ~260,000,000 bases per subset) were assembled using Flye v2.9.5 (6), Raven v1.8.3 (7), and miniasm v0.3 + minipolish v0.1.2 (8). A consensus assembly, involving up to three assemblies per tool, was generated using Trycycler, following the workflow described at https://github.com/rrwick/Trycycler/wiki, including trimming and rotating of circular contigs. The consensus assembly was polished using Medaka v2.0.1 (https://github.com/nanoporetech/medaka), with flag --bacteria. No plasmids were identified with plassembler v1.6.2 (9), using the reads with a length of >1,000bp (reads, 245,987; total bases, 2,325,648,352; N50, 15,022) and the polished Trycycler assembly as inputs. Annotation was performed using the NCBI Prokaryotic Genome Annotation Pipeline v6.8 (10).

The genome consists of a single circular chromosome with a length of 3,090,452 base pairs and a GC content of 40.8%. Using CheckM2 v1.0.2 (11; specific neural network model), the assembly was predicted to be 99.99% complete and free of contamination (0.08% estimated contamination). The genome shares 97.8% average nucleotide identity with *D. formicigenerans* ATCC 27755 (RefSeq assembly GCF_025150245.1), the type strain of the species, and representative genome in the Genome Taxonomy Database release 220 (12). The genome was predicted to contain 3,061 total genes, encoding for 2,907 proteins, 53 tRNAs, and 6 complete sets of rRNA genes.

## Data Availability

The genome sequence has been deposited in the GenBank/DDBJ/EMBL database under accession number CP173381; the version described in this paper is the first version. Sequence data can be found at NCBI’s Sequence Read Archive under accession number: SRR31300268.
